# Clinical Evaluation of Titanium-Alloy Bands for Sternal Closure after Median Sternotomy: Single-Center Experience

**DOI:** 10.1155/2022/8622498

**Published:** 2022-05-29

**Authors:** Feng Qiu, Su Wang, Xianglan Jin, Xiaoyun Wu, Jian Zhou

**Affiliations:** Department of Cardiac Surgery, Shanghai Tenth People's Hospital, School of Medicine, Tongji University, Shanghai 200072, China

## Abstract

**Background:**

Median sternotomy is the most applied approach in open-heart surgery, while potential complications such as postoperative bleeding, sternal dehiscence, and deep sternal wound infection (DWSI) still remain a challenge to cardiac surgeons. Several new sternum-closure products instead of stainless wire have been brought into clinical application. The objective of this retrospective study is to evaluate the novel sternum-fixing product in terms of clinical outcomes.

**Methods:**

689 consecutive cases undergoing cardiac surgery through median sternotomy between February 2015 and December 2018 in our center were enrolled in this study. All the cases were divided into two groups according to different sternum fixation methods: wire cerclage group and rigid fixator group. The demographic as well as clinical data including the mediastinal drainage of first, second, and third post-op 24 hours, the total mediastinal drainage of post-op 72 hours, ICU duration, length of hospital stay, and post-op mortality in 30 days were collected and compared between the two groups.

**Results:**

278 cases were enrolled in the wire cerclage group and 411 cases in the rigid fixator group. There is no significant difference in the demographic data between the two groups, while the mediastinal drainage in the first and third 24 hours after surgery and the total mediastinal drainage in postoperative 72 hours of the rigid fixator group were significantly less than those of the wire cerclage group (*P* < 0.05). No significant difference was found in other clinical outcomes between the groups including ICU duration, LOS in hospital, and 30-day mortality. 14 cases (5.0%) in the wire cerclage group and 11 cases (2.7%) in the rigid fixator group had sternotomy-related complications including severe postoperative bleeding, sternal dehiscence, and DSWI.

**Conclusion:**

Compared with the conventional wire cerclage, the new rigid fixator is superior in median sternotomy closure in terms of postoperative mediastinal bleeding as well as incidence of sternotomy-related complications.

## 1. Introduction

Although several mini-invasive approaches have been applied in adult cardiac surgery, median sternotomy remains the most common approach in cardiac surgery all over the world. The sternal closure after median sternotomy has been a challenge to cardiac surgeons because the postoperative sternal-related complications such as severe retrosternal bleeding, sternal dehiscence, and deep sternal wound infection (DSWI) would lead to prolonged length of stay in hospital, increased medical costs during hospitalization, impacted life quality, or even higher mortality after surgery [1, 2]. It is reported that the incidence of DSWI could be 0.5%–6% and the mortality rate could be as high as 15% with steel wire cerclage, which is the most applied sternal closure method after median sternotomy [3–6]. Though it is cheap, it is not optimal since the wires cannot provide adequate rigid fixation and have the risk of transversely cutting off the sternum leading to sternal dehiscence, especially in high-risk patients with fragile sternums or malnutrition.

In the past decade, many efforts have been made to develop new sternal closure products, such as rigid titanium plates, polyether ether-ketone (PEEK) bands, and stainless steel multibraided cables [7–11]. Premarket biomechanical studies, as well as benchtop studies, have shown that PEEP bands have superior strength and fatigue resistance to stainless steel wire; moreover, they are less likely to cut through the sternum than wires as they are 5 mm wide. However, the application of PEEP bands still needs the penetration of intercostal space, which increases the risk of injury to intercostal blood vessels. Compared with steel wire and PEEK bands, rigid titanium plates are able to provide optimal approximation and stability after median sternotomy, while the cost associated with the technique remains a drawback and the damage caused by the drill to the sternum makes it unsuitable for the osteoporotic bone [12, 13]. Several small-sized randomized controlled studies, as well as retrospective clinical studies, have shown that compared with conventional wire cerclage, the new materials are superior in sternal fixation in terms of sternal ossification and stability [14–16], while its adoption by the surgeons has been limited mainly because of the well-established experience, better availability, and lower cost of the steel wire.

A new rigid peristernal band made of titanium alloy was introduced to our center in February 2015. Since then, the sternum closure protocol has gradually been adopted from wire cerclage in a ‘figure-of-eight' manner to the peristernal band, which makes sternum closure easier and faster during the operation. The objective of this retrospective study is to determine whether the rigid peristernal band is superior to steel wire cerclage in preventing the sternal-related complications after median sternotomy.

## 2. Materials and Methods

Patients who underwent cardiovascular surgery through middle sternotomy in the Department of Cardiothoracic Surgery, Shanghai, Tenth People's Hospital, from February 2015 to December 2018 were enrolled in this study.

### 2.1. Inclusion Criteria

Inclusion criteria were as follows: (1) patients aged between 18 years and 85 years; (2) patients with no previous history with middle sternotomy or other chest surgeries.

### 2.2. Exclusion Criteria

Exclusion criteria were as follows: (1) patients aged below 18 years or over 85 years; (2) patients with application of more than one sternum fixation method during surgery; (3) patients with long-term oral corticosteroid usage; (4) patients with congenital sternum or thoracic deformities; (5) patients with a history of chest surgery, such as extended radical mastectomy; (6) patients with a loading dose of DOATs within 72 hours before surgery; (7) patients with an unexpected secondary operation due to nonsternal reasons.

### 2.3. Ethics

This study complies with medical ethics standards and has been approved by the Hospital Ethics Committee (approval number: SHSYIEC-KY-4.0/16-31/01). Given the retrospective nature of the data, individual patient's written informed consent was waived.

### 2.4. Data Collection

Demographic data including gender, age, height, and body weight of all enrolled patients were collected. Besides, clinical data such as comorbidities (hypertension and diabetes), sternum fixation methods (stainless steel wire or titanium-alloy bands) and the number of devices used, the application of cardiopulmonary bypass (CPB) during surgery, perioperative left ventricular ejection fraction (LVEF), the volume of mediastinal drainage in the first, second, and third 24 hours postoperatively, the incidence of sternal-related complications including retrosternal hemorrhage, sternal dehiscence and DSWI, duration of ICU, length of hospital stay, and 30-day mortality after surgery were also collected.

### 2.5. Research Methods

This is a nonrandomized retrospective clinical study. Based on the method of sternum fixation, the enrolled patients were divided into two groups: the steel wire group and the titanium-alloy band group. The demographic data such as age, gender, BMI, surgical procedures, and comorbidities between the two groups were compared. The evaluation of two different sternal fixation methods was executed by comparing the volume of postoperative mediastinal drainage, incidence of sternal-related complications, and other clinical outcomes.

### 2.6. Statistical Method

SPSS 19.0 software was applied for statistical analysis. The measurement data of normal distribution were expressed as mean ± SD, and the *t*-test was used for the comparisons between the groups. Nonnormal distribution measurement data were expressed by M(Q1, Q3), and the Wilcoxon test was used for comparison between the groups. Counting data were obtained using the chi-square test or Fisher's exact probability. A *P* value of <0.05 was considered to be statistically significant.

## 3. Results

A total of 689 patients who underwent open-heart surgery through median sternotomy by the same surgical team in our center from February 2015 to December 2018 were enrolled in this study, with an average age of 61.5 years and an average BMI of 24.2. Among them, 450 patients were male, accounting for 65.3%. The demographic data have been shown in Table 1. Coronary artery bypass grafting (315 cases) and heart valve surgery (214 cases) are the two most common procedures. Other procedures included complex cardiac surgeries: CABG combined with valvular surgery (39 cases), surgeries for congenital heart disease (26 cases), ascending aortic surgery (37 cases), and other cardiac surgeries (22 cases). Cardiopulmonary bypass was applied in 466 cases during surgery. Hypertension and type 2 diabetes were diagnosed in 52.4% (361 cases) and 23.1% (159 cases) of all patients, respectively.

### 3.1. Operation Method

**Figure 1:**
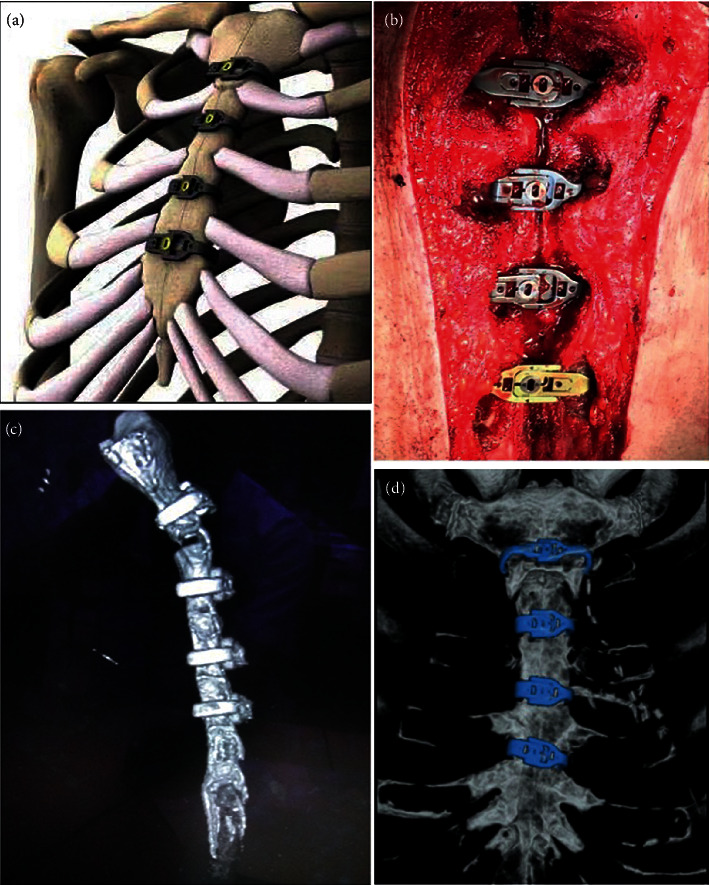
Schematic of the titanium-alloy sternal fixation system. (a) Rendering of sternal bands. (b) Four sternal bands inserted into the intercostal space. (c, d) Thoracic CT scan and sternum reconstruction 90 days after surgery showing satisfactory healing of the sternum.

A mediastinal drainage tube, as well as a pericardial drainage tube, was routinely placed during surgery. A 30Fr mediastinal drainage tube was placed at the deep sternal space, and the tip of the drainage tube is located at the level of the first intercostal space; the pericardial drainage tube is 28Fr, which was placed in the lowest part of pericardial cavity (next to the diaphragm). The number of applied stainless steel wires or titanium-alloy bands mainly depended on the patient's bodyweight. For patients below 65 kg, the sternum was fixed with three wires in a figure-of-eight manner or three titanium-alloy bands. For patients with bodyweight over 65 kg, four steel wires or titanium-alloy bands were used. All the sternal bands were inserted and locked in the intercostal space, without the necessity of penetrating the intercoastal space (Figure 1). Before the placement of the sternal bands, the target intercoastal space (usually from 1^st^ to 4^th^) needed to be exposed and the width and the height of the sternum were measured using a special tool in order to select the sternal bands with appropriate size. Then, the two halves of the sternal bands were fixed on the carrier and inserted into the intercoastal space. As the last step, the sternal bands were closed at a proper tension and locked using the special screwdriver.

### 3.2. Demographic Data between the Groups

All the enrolled cases were divided into two groups depending on the method of sternum fixation: 278 cases in the steel wire group and 411 cases in the titanium band group. The demographic data between the two groups have been shown in Table 2. The ratio of male to female patients in the steel wire group was 1.65 : 1, the average age was 61.1 years, and the average BMI was 23.9. The proportions of patients with hypertension and diabetes were 54.0% and 21.6%, respectively. The proportion of patients with intraoperative cardiopulmonary bypass application was 64.0%. The proportion of male patients in the titanium band group was 2.08 : 1, the average age was 61.7 years, and the average BMI was 24.6. The proportions of patients with hypertension and diabetes were 51.3% and 24.1%, respectively. The proportion of extracorporeal circulation used during surgery was 70.3%. There was no significant difference between the two groups in comparison with the above data (*P* > 0.05).

### 3.3. Comparison of Clinical Data between the Groups

The preoperative and postoperative LVEF of the steel wire group were 0.61 ± 0.1 and 0.60 ± 0.09, respectively; the preoperative and postoperative LVEF of the titanium band group were 0.60 ± 0.09 and 0.59 ± 0.08, respectively. There was no significant difference between the two groups (Table 3).

The volume of mediastinal drainage in the first 24 hours after surgery was 469.7 ± 337.4 ml in the steel wire group and 361.5 ± 242.6 ml in the titanium band group (*P* < 0.001); the mediastinal drainage volume in the second 24 hours after surgery was 265.1 ± 171.0 ml in the steel wire group and 242.0 ± 130.6 ml in the titanium band group (*P* = 0.053); the mediastinal drainage volume in the third 24 hours after surgery was 159.7 ± 143.2 ml in the steel wire group and 133.1 ± 110.2 ml in the titanium band group (*P* = 0.014); the total amount of mediastinal drainage in the 72 hours after the operation was 840.3 ml ± 477.2 ml in the steel wire group and 692.4 ± 395.6 ml in the titanium band group (*P* < 0.001). There were no significant statistical differences in the mediastinal drainage between the two groups at all observed time points only except for the second postoperative 24 hours.

The duration of ICU was 81.3 ± 55.2 hours and 93.4 ± 75.6 hours in the steel wire group and the titanium band group, respectively (*P* = 0.037). The total hospital stay of the steel wire group was 27.9 ± 24.9 days, with no statistically significant difference compared with the titanium band group (24.5 ± 26.8 days, *P* = 0.067). The postoperative 30-day mortalities of both groups were 5.4%, with no significant difference (*P* = 0.21).

### 3.4. Clinical Data of Patients with Postoperative Sternum Complications

As a result, 25 cases required second surgery due to poor sternal union or retrosternal hemorrhage with an incidence of 5.1%. Among them, there were 18 males and 7 females, with an average age of 65.5 ± 12.6 years and an average BMI of 26.2 ± 3.7. Coronary artery bypass grafting is the major procedure (13 cases). 19 patients had hypertension, and 7 patients had diabetes. Cardiopulmonary bypass was applied in 76% of these surgeries (19 cases). The data have been shown in Table 4. The first 24-hour drainage volume of the abovementioned patients with secondary operations was 738.3 ± 578.5 ml, the average duration of ICU was 109 ± 41 hours, and the average hospital stay was 60.5 ± 70.5 days. Three patients died within 30 days after surgery, with a mortality rate of 12%.

Among the abovementioned 25 patients undergoing secondary operations, there were 14 cases in the steel wire group with an incidence rate of 5.0% and 11 cases in the titanium band group with an incidence rate of 2.7%. There was no significant difference between the two groups (*P* = 0.172). The procedure, comorbidities, and clinical data are detailed in Table 5. There is no significant difference between all data groups.

## 4. Discussion

Sternal fixation after median sternal incision heart surgery is very important for postoperative recovery. The sternum fixation technique should provide precise sternal alignment and proper tension to ensure bone healing. The classic stainless wire technology is widely used all over the world because of the highest availability and economical efficiency. However, it is not an ideal choice, since the cutting of sternum caused by the wire and the risk of poststernal bleeding account for a large part of second thoracotomy after cardiac surgery, regardless of interrupted suture or figure-of-8 suture. The “Robicsek's weave” technique can effectively reinforce the fragile sternum and reduce the cutting of the sternum by the wire. It is often used in patients who have undergone second sternal resection or who have asymmetrical sternal cuts. However, the suture process is complicated and more time-consuming, accompanied by the risk of postoperative bleeding. From a prospective randomized controlled trial, Allen KB et al. found that wire cerclage is the only predictor of sternal complications after median sternal incision [17]. In recent years, rigid sternal plates, PEEK bands, and other new types of sternal fixation materials have been introduced in clinical application. Several relevant clinical studies have confirmed their advantages in reducing the incidence of poor sternal healing after cardiac surgery with median sternal incision. The use of enhanced sternal stabilization to potentially reduce the risk of sternal infections, particularly in high-risk patients, recently, was recognized by the STS Practice Guidelines and an expert consensus by Lazar et al., where it was given a Level IIB recommendation [18].

**Figure 2:**
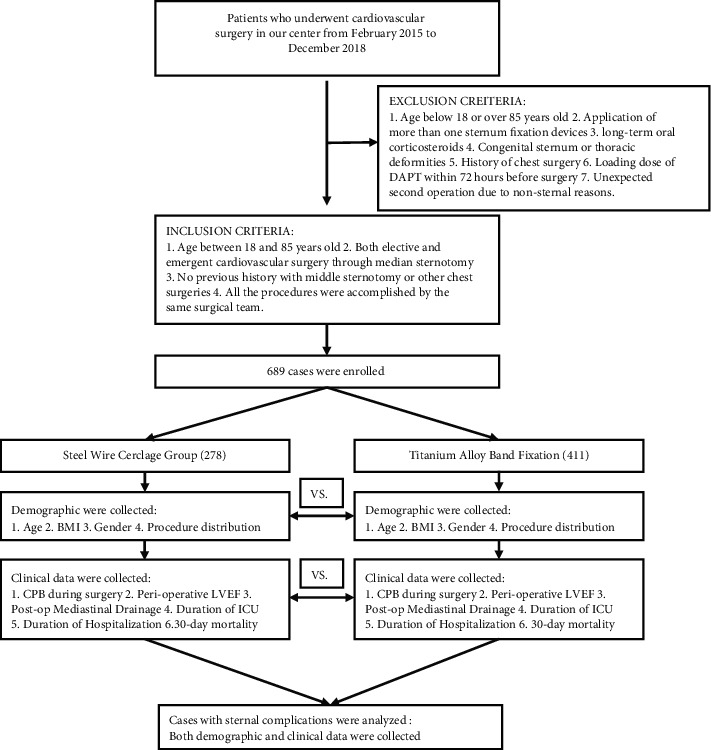
The flow chart showing the design of this retrospective clinical study.

Our center has been using Watson titanium-alloy sternum fixation bands since 2017. There are several advantages of this new product, such as wider width than in steel wires that significantly reduced the cutting force on the sternum and localized in the intercostal space with no damage to the sternum compared to the rigid plates. Furthermore, there is no need to penetrate the intercostal space, which may dramatically reduce the risk of intercostal vascular injury and retrosternal bleeding (Figure 2).

After retrospective analysis of 689 patients undergoing median sternal incision heart surgery in our center, the results have shown that the mediastinal drainage in the first 24 hours after surgery and the total mediastinal drainage in the 72 hours after surgery in the sternal band fixation group were significantly less than those in the steel wire fixation group, which can be attributed to the lower risk of injuring the intercostal blood vessels when sternal bands were used. In addition, the rate of secondary operations in the sternal band fixation group due to postoperative mediastinal bleeding or poor sternal healing was 2.7%, compared with the 5.0% in the steel wire group, while the difference did not reach statistical significance due to the limited number of cases.

In clinical practice, we believe that another advantage of the titanium-alloy sternal band compared to the steel wire is that it significantly reduces the cutting of the sternum. This is, particularly, important for elderly female patients, who often suffered with serious sternal osteoporosis and thus are at higher risk of sternal cutting caused by the wire fixation. Among the 14 second thoracotomy patients in the steel wire fixation group, there were 9 cases which were due to sternal dehiscence. There were even three patients who had to receive a third operation to achieve sternal healing. In contrast, among the 11 secondary-operation patients in the sternal band group, only 3 patients suffered from sternal dehiscence. Other reasons for the secondary operation include poor sternal alignment and severe pain caused by the displacement of the sternal bands, which were observed in 2 patients.

It is necessary to notice that all of the five cases occurred in early clinical use of the sternal bands, which could be attributed to the inherent learning curve. In our experience, attention should be paid to two aspects in the clinical setting: adding adequate closing force to the bands based on the firmness of the sternum, as well as selecting the titanium band with proper size to avoid the mismatch between the height of the bands and the thickness of the sternum and subsequent severe postoperative chest pain or even sternal dehiscence. In addition, there are also some clinical situations that titanium bands are not appropriate for use, such as the abnormal fusion of intercostal spaces and severe asymmetric sternal cut, when the “Robicsek's weave” using the steel wire or rigid plate fixation is preferred. Since the second half of 2017, titanium bands have gradually replaced steel wires as the main sternum fixation method in our center. With the increase in experience, the incidence of sternal complications with titanium-alloy bands has been significantly reduced.

### 4.1. Shortcomings of the Study

Because it is a single-center nonrandomized controlled retrospective study, the results of this study need to be further verified in prospective randomized controlled clinical trials. In addition, this study included continuous data from the same center over a period of time. However, compared with classic steel wire fixation method, the learning curve of the novel sternal bands played an important role in relatively poor clinical outcomes of the sternal band fixation group and thus to a certain extent weakens the advantages of sternal bands compared to steel wires.

## 5. Conclusions

As a single-center experience, we have confirmed through this retrospective clinical study that new sternal fixation techniques, such as titanium-alloy sternal bands, have significant advantages over steel wires in sternal fixation after cardiac surgery with a median sternotomy, including reducing the risk of poststernal bleeding and poor sternal healing. As more and more new sternal fixation devices are applied in clinical settings, sternal-related complications have been continuously decreased, making cardiac surgery with median sternotomy safer than ever before [17, 18].

## Figures and Tables

**Table 1:** Demographic data.

Total no. of cases		689
Age (mean ± SD)		61.5 ± 11.4
BMI (mean ± SD)		24.2 ± 4.1
Gender	Male	450
	Female	239

Procedures	CABG	351
	Valvular	214
	CABG + valvular	39
	Congenital heart disease	26
	Aortic	37
	Others	22

Comorbidities (%)	Hypertension	361 (52.4%)
	Type 2 diabetes	159 (23.1%)
Surgeries with CPB (%)		466 (67.6%)

**Table 2:** Comparisons of demographic data and procedure distribution between the two groups.

		Stainless wire group	Titanium band group
No. of cases		278	411
Age (mean ± SD)		61.1 ± 12.4	61.7 ± 10.6
BMI (mean ± SD)		23.9 ± 3.6	24.6 ± 3.5
Gender	Male	173	277
	Female	105	134

Procedures	CABG	133	218
	Valvular	98	116
	CABG + valvular	17	22
	Congenital heart disease	11	11
	Aortic	13	28
	Others	6	16

Comorbidities (%)	Hypertension	150 (54.0%)	211 (51.3%)
	Type 2 diabetes	60 (21.6%)	99 (24.1%)

Surgeries with CPB (%)		178 (64.0%)	289 (70.3%)

There was no statistical significance when comparing all the data given between the two groups.

**Table 3:** Comparisons of clinical outcomes between the two groups.

		Stainless wire group	Titanium band group
LVEF	Preoperative	0.61 ± 0.10	0.60 ± 0.09
	Postoperative	0.60 ± 0.09	0.59 ± 0.08

Post-op mediastinal drainage (ml)	First 24 hours	469.7 ± 337.4*∗*	361.5 ± 242.6*∗*
	Second 24 hours	265.1 ± 171.1	242.0 ± 130.6
	Third 24 hours	159.7 ± 143.2^†^	133.1 ± 110.2^†^
	Total volume	840.3 ± 477.2^‡^	692.4 ± 395.6^‡^

Duration of ICU stay (hour)		81.3 ± 55.2¶	93.4 ± 75.6¶
Duration of hospitalization (day)		27.9 ± 24.9	24.45 ± 26.8
Mortality in 30 post-op day		5.4%	5.4%

*∗P* < 0.001, †*p* = 0.013, ‡*P* < 0.001, and ¶*p* = 0.037 for the stainless wire group compared with the titanium-alloy band group.

**Table 4:** Demographic and clinical outcomes of patients undergoing second surgeries due to sternal complications.

Total no.		25
Age (mean ± SD)		65.5 ± 12.6
BMI (mean ± SD)		26.2 ± 3.7

Gender	Male	18
	Female	7

Procedures	CABG	13
	Valvular	6
	CABG + valvular	3
	Congenital heart disease	0
	Aortic	3
	Others	0

Comorbidities	Hypertension	19
	Type 2 diabetes	7

Surgeries with CPB		19

LVEF	Preoperative	0.59 ± 0.09
	Postoperative	0.59 ± 0.05

Post-op mediastinal drainage (ml)	First 24 hours	738.3 ± 579.5
	Second 24 hours	317.2 ± 173.8
	Third 24 hours	170.0 ± 100.6

Duration of ICU stay (hour)		109.4 ± 41.5
Duration of hospitalization (day)		60.5 ± 70.5
Mortality in 30 post-op day		12

**Table 5:** Comparison of cases undergoing second surgery in two groups.

		Stainless wire group	Titanium band group
Total no.		14 (5.0%)	11 (2.7%)
Age (mean ± SD)		66.9 ± 12.7	63.7 ± 12.8
BMI (mean ± SD)		26.1 ± 3.2	26.4 ± 4.5
Gender	Male	11	7
	Female	3	4
Procedures	CABG	9	4
	Valvular	2	1
	CABG + valvular	1	5
	Congenital heart disease	0	0
	Aortic	2	1
	Others	0	0
Comorbidities (%)	Hypertension	11	8
	Type 2 diabetes	4	3
Surgeries with CPB (%)	10	9	
LVEF	Preoperative	0.59 ± 0.1	0.59 ± 0.07
	Postoperative	0.58 ± 0.05	0.60 ± 0.06
Post-op mediastinal drainage (ml)	First 24 hours	851.0 ± 580.4	605.2 ± 574.1
	Second 24 hours	286.6 ± 155.1	354.3 ± 195.7
	Third 24 hours	163.2 ± 85.8	176.4 ± 116.3
Duration of ICU stay (hour)		116 ± 45.8	102 ± 37.0
Duration of hospitalization (day)		72.5 ± 89.1	46.4 ± 38.7
Mortality in 30 post-op day		14%	9%

## Data Availability

The data used to support the findings of this study are available from the corresponding authors upon request.

## Abbreviations

BMI: Body mass index
CABG: Coronary artery bypass grafting
CHD: Congenital heart disease
CPB: Cardiopulmonary bypass
DAPT: Dual antiplatelet therapy
DSWI: Deep sternal wound infection
LVEF: Left ventricular ejection fraction
PEEK: Polyether ether-ketone
